# Immunization strategies for individuals with sickle cell anemia: A narrative review

**DOI:** 10.1097/MD.0000000000039756

**Published:** 2024-09-20

**Authors:** Emmanuel Ifeanyi Obeagu, Getrude Uzoma Obeagu

**Affiliations:** a Department of Medical Laboratory Science, Kampala International University, Uganda; b School of Nursing Science, Kampala International University, Uganda.

**Keywords:** immunity, immunization, sickle cell anemia

## Abstract

Sickle cell anemia (SCA) is a hereditary blood disorder characterized by the production of abnormal hemoglobin, leading to the formation of sickle-shaped red blood cells. These distorted cells can obstruct blood flow, causing vaso-occlusive crises and increasing the risk of severe infections due to functional asplenia and immune system dysregulation. Immunization is a crucial strategy to mitigate infection-related complications in individuals with SCA, necessitating a comprehensive and tailored vaccination approach. Current immunization guidelines for individuals with SCA recommend a combination of standard and additional vaccines to address their heightened susceptibility to infections. Key vaccines include pneumococcal conjugate (PCV13) and polysaccharide (PPSV23) vaccines, meningococcal conjugate (MenACWY) and serogroup B (MenB) vaccines, *Haemophilus influenzae* type b (Hib) vaccine, annual influenza vaccine, and hepatitis A and B vaccines. These vaccinations aim to provide broad protection against pathogens that pose significant risks to patients with SCA. Despite generally adequate immune responses, the variability in vaccine efficacy due to immune dysfunction necessitates booster doses and additional vaccinations. This narrative review highlights the importance of adhering to current immunization recommendations and addresses challenges such as access to care, vaccine hesitancy, and monitoring vaccination status.

## 1. Introduction

Sickle cell anemia (SCA) is a genetic blood disorder resulting from a single nucleotide mutation in the beta-globin gene, leading to the production of abnormal hemoglobin known as hemoglobin S. Under low oxygen conditions, hemoglobin S polymerizes, causing red blood cells to deform into a characteristic sickle shape. These sickle-shaped cells can obstruct blood flow in small blood vessels, leading to painful vaso-occlusive crises, chronic hemolysis, and multiorgan damage. Beyond these complications, individuals with SCA have an increased susceptibility to infections due to functional asplenia and other immune system abnormalities.^[1]^ The increased infection risk in individuals with SCA is a major contributor to morbidity and mortality. Functional asplenia, typically developing in early childhood, significantly impairs the body’s ability to clear encapsulated bacteria, such as *Streptococcus pneumoniae*, *Haemophilus influenzae*, and *Neisseria meningitidis*. Additionally, chronic hemolysis and resultant endothelial dysfunction contribute to immune dysregulation, further exacerbating infection risks. Therefore, preventive measures, particularly immunization, are vital for managing SCA and improving patient outcomes.^[2]^ Immunization is one of the most effective public health interventions, reducing the incidence and severity of infectious diseases. For individuals with SCA, the benefits of vaccination are particularly pronounced due to their heightened vulnerability to infections. Standard childhood vaccination schedules are essential, but additional vaccines and modified schedules are often necessary to provide adequate protection against specific pathogens that pose increased risks for patients with SCA.^[3]^ Pneumococcal infections are a significant concern for individuals with SCA. The introduction of pneumococcal conjugate vaccines (PCV13) and pneumococcal polysaccharide vaccines (PPSV23) has substantially reduced the incidence of invasive pneumococcal disease in this population. These vaccines are recommended at specific intervals to ensure broad coverage against various pneumococcal serotypes. Despite these measures, breakthrough infections can occur, highlighting the need for continued vigilance and possibly additional booster doses.^[4]^

Meningococcal disease, caused by *N meningitidis*, is another critical infection risk for individuals with SCA. Meningococcal conjugate vaccines (MenACWY) and serogroup B meningococcal vaccines (MenB) are recommended to provide comprehensive protection. The timing and frequency of these vaccinations may be adjusted based on individual risk factors and local epidemiology, ensuring optimal protection against this life-threatening infection.^[5]^ *H influenzae* type b (Hib) vaccination is part of the routine immunization schedule for infants and young children. However, for individuals with SCA, additional doses may be warranted to maintain adequate immunity. Hib infections, although less common in regions with high vaccination coverage, can still pose a significant threat to individuals with SCA due to their compromised immune systems.^[6]^ Influenza remains a substantial risk for individuals with SCA, as the viral infection can exacerbate underlying conditions and trigger severe complications such as acute chest syndrome. Annual influenza vaccination is recommended for all individuals with SCA to mitigate these risks. The effectiveness of the influenza vaccine can vary each season, underscoring the importance of yearly immunization and monitoring for influenza outbreaks.^[7]^ Hepatitis B and hepatitis A vaccinations are also important for individuals with SCA. Chronic liver disease is a potential complication in SCA patients due to iron overload from frequent blood transfusions and other factors. Vaccination against hepatitis B and A can prevent further liver damage and complications associated with these viral infections. Ensuring complete vaccination series and considering accelerated schedules may be necessary for optimal protection.^[5]^ Human papillomavirus (HPV) vaccination is recommended for adolescents and young adults, with particular importance for those with SCA. Immunocompromised individuals, including those with SCA, may have an increased risk of HPV-related complications. Completion of the HPV vaccine series can prevent infections that may lead to cervical and other cancers, significantly improving long-term health outcomes.^[6]^ Despite the clear benefits of immunization, challenges remain in achieving high vaccination coverage in individuals with SCA. Barriers such as access to health care, vaccine hesitancy, and lack of awareness about the importance of additional vaccines need to be addressed. Health care providers play a crucial role in educating patients and caregivers, ensuring timely vaccinations, and implementing reminder systems for booster doses.^[7]^ These guidelines are designed to ensure that individuals with SCA receive appropriate vaccinations at the right time. Here are key immunization guidelines for SCA.^[7]^

### 1.1. Routine childhood vaccinations

○ Diphtheria, tetanus, and pertussis: Administer diphtheria, tetanus, and pertussis vaccines according to the standard childhood vaccination schedule. These vaccines protect against these bacterial infections.^[8]^
○ Hib: Ensure that children with SCA receive the recommended Hib vaccinations. Hib can cause severe illnesses, such as meningitis and pneumonia.^[9]^
○ PCV13: PCV13 protects against certain strains of pneumococcal bacteria. Children with SCA should receive this vaccine to reduce the risk of pneumonia, blood infections, and meningitis.^[10]^
○ Hepatitis B: Administer the hepatitis B vaccine to infants according to the standard schedule. Individuals with SCA are at increased risk of hepatitis B.^[11]^

### 1.2. Annual influenza vaccination

Influenza (flu) vaccination is crucial for individuals with SCA. They are at a higher risk of severe flu-related complications, including acute chest syndrome. Ensure that they receive an annual flu shot.^[12]^

### 1.3. Pneumococcal vaccines

○ PCV13: Administer PCV13 to children with SCA according to the recommended schedule, as it provides protection against certain pneumococcal strains.^[13]^
○ PPSV23: After the age of 2 years, individuals with SCA should receive the PPSV23 vaccine to provide additional protection against pneumococcal infections.^[14]^

### 1.4. Special considerations

○ Meningococcal vaccines: Depending on age and specific risk factors, individuals with SCA may need meningococcal vaccinations. Meningococcal infections can be severe and potentially life-threatening.^[15]^
○ Hib vaccine: Ensure that young children receive the Hib vaccine to prevent infections that can lead to serious complications in individuals with SCA.^[16]^

### 1.5. Travel vaccinations

If individuals with SCA plan to travel internationally, consult with a health care provider for advice on additional vaccinations, such as those required for specific destinations.^[17]^

### 1.6. Booster shots and catch-up vaccinations

Stay up-to-date with booster shots and catch-up vaccinations, especially for individuals who may have missed recommended vaccines earlier in life.^[18]^

### 1.7. Pain management during vaccination

Recognize that individuals with SCA may have a lower pain threshold and may be more sensitive to vaccine injections. Employ strategies for minimizing pain and discomfort during vaccination, such as using smaller needles or applying topical numbing agents.^[19]^ It is essential for health care providers to educate individuals with SCA and their caregivers about the importance of immunizations in managing the disease. Regular communication and close monitoring of vaccination status are critical to ensuring individuals with SCA receive the necessary vaccines to protect their health and reduce the risk of infections and complications.^[20]^

## 2. Special considerations in immunization of SCA

Immunizing individuals with SCA requires special considerations due to their increased susceptibility to infections and the unique challenges presented by this condition. Here are the key special considerations to keep in mind when immunizing individuals with SCA.^[14]^

### 2.1. Meningococcal vaccines

Meningococcal vaccines are a crucial component of immunization strategies, particularly for individuals at an increased risk of meningococcal disease. *N meningitidis*, the bacterium responsible for this potentially life-threatening infection, can cause meningitis and septicemia, with rapid onset and severe consequences. There are several types of meningococcal vaccines designed to protect against different serogroups of the bacteria.

#### 2.1.1. Conjugate vaccines

##### MenACWY (Menactra, Menveo):

This vaccine provides protection against 4 major serogroups of *N meningitidis*: A, C, W, and Y. It is routinely recommended for adolescents, college students living in dormitories, and other high-risk groups.

##### MenB (Bexsero, Trumenba):

These vaccines target serogroup B of *N meningitidis*, which has been responsible for a significant proportion of cases in some regions. MenB vaccines are recommended for high-risk populations, including individuals with complement deficiencies, those with asplenia, and certain college populations.

#### 2.1.2. Polysaccharide vaccines

##### MenACWY-D (Menomune):

This vaccine is a polysaccharide vaccine that covers serogroups A, C, W, and Y. It is generally used in specific situations, such as during outbreaks or for individuals older than 55 years.

#### 2.1.3. Timing of vaccination

Meningococcal vaccines are often administered during adolescence, given the higher incidence of the disease in this age group. Catch-up vaccinations may be recommended for certain individuals who missed routine adolescent doses. Individuals at an increased risk of meningococcal disease include those with functional asplenia, complement deficiencies, certain genetic conditions, and those living in close quarters like college dormitories. Depending on the vaccine type, booster doses may be recommended to maintain immunity, especially for those at a sustained higher risk. Meningococcal disease varies by region, and vaccination recommendations may differ accordingly. Understanding the prevalent serogroups in a specific geographic area is crucial for effective immunization strategies.

#### 2.1.4. Combination vaccines

Some vaccines, like MenACWY, may be included in combination with other routine vaccines to streamline the vaccination process and improve coverage.

### 2.2. Pneumococcal vaccines

Individuals with SCA are particularly vulnerable to infections, and pneumococcal vaccines play a crucial role in reducing the risk of severe pneumococcal disease in this population. Pneumococcal vaccines target *S pneumoniae*, a bacterium that can cause serious respiratory and systemic infections, including pneumonia, meningitis, and septicemia. Individuals with SCA have an increased susceptibility to infections due to functional asplenia, a condition where the spleen does not function effectively. As the spleen plays a vital role in defending against certain bacterial infections, including those caused by *S pneumoniae*, individuals with SCA are at a heightened risk of pneumococcal diseases. There are 2 main types of pneumococcal vaccines: PCV and PPSV. The PCV is often recommended for children, while the PPSV is typically administered to adults. These vaccines cover different serotypes of *S pneumoniae*, providing a broad spectrum of protection against pneumococcal infections. Immunization guidelines for individuals with SCA emphasize the importance of pneumococcal vaccination. Children with SCA should receive the recommended doses of PCV according to the standard immunization schedule. Additionally, adults with SCA are advised to receive PPSV, with some guidelines recommending revaccination after a certain interval to maintain protective immunity. Pneumococcal vaccines stimulate the immune system to produce antibodies against specific serotypes of *S pneumoniae*. By inducing an immune response, these vaccines help prevent invasive pneumococcal disease and reduce the severity of infections in case exposure occurs. Despite the importance of pneumococcal vaccination in individuals with SCA, challenges such as vaccine hesitancy, access to health care, and patient education may impact vaccine uptake. Health care providers should address these challenges by implementing strategies to improve awareness, accessibility, and adherence to vaccination schedules.

### 2.3. Hib vaccine

The Hib vaccine is an essential component of the immunization strategy for individuals with SCA. Hib is a bacterium that can cause severe infections, including meningitis, pneumonia, and septicemia. Given the increased susceptibility of individuals with SCA to bacterial infections, particularly encapsulated bacteria, the Hib vaccine plays a crucial role in preventing potentially life-threatening complications. Individuals with SCA, due to functional asplenia and compromised immune function, are at an elevated risk of infections caused by encapsulated bacteria, including Hib. Hib infections can be severe and lead to life-threatening conditions, making immunization a critical preventive measure. The Hib vaccine is designed to stimulate the immune system to produce antibodies against the Hib bacterium. By inducing an immune response, the vaccine helps protect against invasive Hib disease and its associated complications. Immunization guidelines for individuals with SCA typically include the administration of the Hib vaccine as part of the routine childhood immunization schedule. It is often given in a series of doses, starting in infancy. Catch-up vaccinations may be recommended for older individuals who may have missed the initial doses. The Hib vaccine is crucial for boosting immunity against Hib, thereby preventing invasive diseases. The vaccine contributes to the overall protection of individuals with SCA, reducing the risk of severe infections that could further complicate their health status. While the Hib vaccine is a vital preventive measure, challenges such as vaccine hesitancy, access to health care, and awareness may impact vaccine uptake. Health care providers must address these challenges by implementing strategies to enhance education, promote accessibility, and ensure adherence to vaccination schedules.

### 2.4. Catch-up vaccinations

The vaccination schedule for individuals with SCA is important for preventing infections and complications associated with the weakened immune system in this population. The recommended vaccinations for individuals with sickle cell disease (SCD) typically follow guidelines set by health authorities and may include catch-up doses if vaccinations were missed during childhood. Vaccination against *N meningitidis* is important to prevent meningitis and other invasive infections. Different serogroups are covered by various meningococcal vaccines. Annual influenza vaccination is recommended to protect against seasonal flu viruses. If not previously vaccinated, individuals with SCD may be recommended to receive the hepatitis B vaccine. If individuals have not had chickenpox or have not been vaccinated against varicella, catch-up vaccination may be recommended. Catch-up doses of the Measles, Mumps, and Rubella vaccine may be recommended if individuals with SCD did not receive the standard doses during childhood.

### 2.5. Pain management during vaccination

Recognize that individuals with SCA often have a lower pain threshold and are more sensitive to needle injections. Employ pain management strategies, such as using smaller gauge needles, applying topical numbing agents, and ensuring proper technique to minimize discomfort during vaccination.

### 2.6. Annual influenza vaccination

Emphasize the importance of annual influenza (flu) vaccination for individuals with SCA, as they are at a higher risk of severe flu-related complications. Encourage timely vaccination before the flu season begins.

### 2.7. Communication and education

Health care providers should actively engage in education and communication with individuals with SCA and their caregivers. Discuss the rationale for each vaccination, potential side effects, and the importance of staying up-to-date with immunizations.

### 2.8. Monitoring and surveillance

Keep careful records of immunizations and vaccination schedules for individuals with SCA. Ensure regular follow-up to monitor vaccination status and address any concerns or adverse reactions promptly.

### 2.9. Individualized care

Recognize that the immunization needs of individuals with SCA may vary based on age, overall health, and other individual factors. Tailor vaccination strategies to meet the specific needs of each patient.

### 2.10. Multidisciplinary approach

Collaboration between hematologists, immunologists, and primary care physicians is essential to provide comprehensive care for individuals with SCA. A multidisciplinary approach ensures that immunization strategies are integrated into the overall management of the disease.

Special considerations in immunizing individuals with SCA require a proactive and individualized approach to address their unique vulnerabilities and health care needs. These considerations aim to protect this population from infections and complications.^[21]^

## 3. Challenges and barriers of immunization in SCA

Immunization in individuals with SCA can be challenging due to several factors, including the unique health considerations and complications associated with this condition. Here are some of the challenges and barriers to immunization in individuals with SCA^[16]^:

1. Weakened immune system: It is important to note that individuals with SCD may be more susceptible to infections, which can impact their overall immune system. SCD is a genetic disorder that affects hemoglobin, the protein in red blood cells that carries oxygen. The altered hemoglobin can cause the red blood cells to become rigid and take on a sickle shape, leading to various complications, including increased susceptibility to infections. SCD can lead to functional asplenia, where the spleen is unable to effectively filter and remove bacteria from the bloodstream. This increases the risk of bacterial infections, particularly those caused by encapsulated bacteria such as *S pneumoniae*. SCD can impair the immune response, making it more difficult for the body to fight off infections. This may be related to the chronic inflammation and oxidative stress associated with the disease. The chronic anemia associated with SCD can weaken the overall health of individuals, affecting their ability to mount a robust immune response. Individuals with SCD may also be more susceptible to certain viral infections, including influenza and other respiratory viruses. To address the increased vulnerability to infections, individuals with SCA often receive vaccinations to protect against specific pathogens. Common vaccinations include those against pneumococcal bacteria, Hib, meningococcal bacteria, and influenza. These vaccinations are part of the routine care for individuals with SCD, and catch-up vaccinations may be recommended in certain cases.
2. SCA can lead to a weakened immune system, making individuals more susceptible to infections. This weakened immune response can affect the effectiveness of vaccines, making it important to carefully time and administer immunizations.^[14]^
3. Risk of infection: People with SCA are at a higher risk of infections, including bacterial infections, pneumonia, and sepsis. These infections can be severe and life-threatening, making immunization crucial.^[22]^
4. Sickle cell crisis: SCA is characterized by recurrent sickle cell crises, which can be triggered by infections. Immunizations may need to be carefully scheduled to minimize the risk of triggering a crisis.^[23]^
5. Pain management: SCA patients often experience chronic pain, and the pain associated with immunization injections can be a barrier to compliance. Health care providers must be sensitive to the pain management needs of these patients.^[24]^
6. Limited vaccine response: The spleen, an important organ in the immune system, is often damaged or removed in individuals with SCA. This can impair the response to certain vaccines, particularly those targeting encapsulated bacteria. Alternative vaccination strategies may be needed.^[25]^
7. Access to health care: Socioeconomic factors, lack of access to health care, and health disparities can pose barriers to immunization for individuals with SCA. Ensuring access to health care services, including vaccination clinics, is critical.^[26]^
8. Vaccine hesitancy: Some individuals with SCA and their families may have concerns about vaccines, either due to misinformation or fear of adverse effects. Educating patients and their families about the importance and safety of immunization is essential.
9. Vaccine scheduling: Proper scheduling of vaccines is important for individuals with SCA. Some vaccines may need to be administered earlier or more frequently than in the general population to ensure adequate protection.^[27]^
10. Vaccine selection: Not all vaccines are equally important for individuals with SCA. Health care providers should prioritize vaccines that protect against infections that are particularly dangerous for these patients, such as pneumococcal vaccines.^[28]^
11. Medical records and follow-up: Maintaining accurate medical records and ensuring that patients receive appropriate booster shots and follow-up vaccinations can be a challenge, especially when patients receive care from multiple health care providers.

To overcome these challenges and barriers, it is essential for individuals with SCA to work closely with their health care providers. A multidisciplinary approach that includes hematologists, infectious disease specialists, and primary care physicians is often needed to develop a personalized immunization plan that addresses the unique needs and risks associated with SCA. Education, outreach, and advocacy efforts can also help.^[29]^

## 4. Promoting immunization in SCA

Promoting immunization in individuals with SCA is essential to protect them from infections that can have serious consequences. Here are some strategies to promote immunization in this population.^[30]^

### 4.1. Education and awareness

Educate individuals with SCA and their families about the importance of immunization in preventing infections. Provide information about the specific vaccines recommended for SCA patients and their schedule.

### 4.2. Collaboration with health care providers

Encourage individuals with SCA to establish a strong relationship with health care providers, including hematologists and primary care physicians. Ensure that health care providers are aware of the immunization needs of SCA patients and are proactive in recommending and administering vaccines.

### 4.3. Personalized immunization plans

Work with health care providers to develop personalized immunization plans that consider the individual’s specific health status and medical history. Ensure that immunization schedules are tailored to the needs of the patient and are aligned with their overall health care plan.

### 4.4. Vaccine clinics and access

Advocate for improved access to health care services, including vaccination clinics, for individuals with SCA. Encourage health care facilities to offer immunization services that are convenient and accessible for this population.

### 4.5. Vaccine administration

Address pain management concerns related to immunization by using smaller needles, topical anesthetics, or other pain-reduction techniques. Ensure that vaccines are administered by health care providers experienced in working with individuals with SCA.

### 4.6. Support and resources

Provide support groups and resources for individuals with SCA and their families, where they can share experiences and information about immunization. Connect individuals with SCA to patient advocacy organizations that can provide guidance and support.

### 4.7. Public health campaigns

Collaborate with public health agencies to run campaigns that raise awareness about the importance of immunization in SCA. Use social media, informational brochures, and community events to disseminate information.

### 4.8. School and workplace education

Partner with schools and workplaces to educate staff and peers about the immunization needs of individuals with SCA. Ensure that individuals with SCA are aware of their rights and accommodations related.

## 5. Future directions of immunization in SCA

The future of immunization in individuals with SCA holds promise as medical research and health care practices continue to evolve. Here are some potential future directions for immunization in SCA.^[31]^ Continued research into vaccine development specifically tailored for individuals with SCA. This may involve developing more effective vaccines or new vaccine formulations that provide enhanced protection. The development of personalized vaccination strategies based on a patient’s unique medical history, genetic factors, and disease severity. This may include genetic testing to identify individuals at higher risk for infections and to guide vaccination decisions. Enhanced research to determine the most effective timing and schedules for vaccination in individuals with SCA. This may involve optimizing the timing of booster shots and identifying the ideal age to begin vaccination. Strategies to improve the immune response to vaccines in individuals with SCA. This may include novel adjuvants, immune-enhancing therapies, or approaches to boost vaccine efficacy.

Greater utilization of telemedicine and remote monitoring to ensure individuals with SCA receive timely vaccinations and follow-up care. Telehealth can help bridge geographical and access-related barriers. Ongoing efforts to educate individuals with SCA and their families about the importance of immunization. Increasing awareness can lead to higher vaccine acceptance rates and better adherence to vaccination schedules. Integration of electronic health records and immunization registries to facilitate tracking and monitoring of vaccination status for individuals with SCA. This can improve coordination of care and reduce missed opportunities for vaccination. Research into the specific risks and benefits of vaccines in individuals with SCA. This includes evaluating the potential for vaccines to trigger sickle cell crises and determining the best practices for minimizing these risks. Focused efforts to address health disparities and ensure that individuals with SCA, especially in underserved communities, have equitable access to vaccines and health care services. Preparedness for emerging infectious diseases by rapidly developing and distributing vaccines, especially when new pathogens pose a significant threat to individuals with SCA. Collaboration between hematologists, infectious disease specialists, primary care physicians, and other health care professionals to create comprehensive care plans that include immunization as an integral part. Ongoing monitoring of vaccine safety and the long-term effects of vaccination in individuals with SCA with a focus on ensuring that vaccines do not contribute to complications of the disease.^[27,32–36]^

## 6. Conclusions

Immunization is a critical component of preventive health care for individuals with SCA. Due to their heightened vulnerability to infections resulting from functional asplenia and immune dysregulation, individuals with SCA benefit significantly from a comprehensive immunization strategy that includes both standard and additional vaccines. Key vaccines, such as pneumococcal conjugate (PCV13), pneumococcal polysaccharide (PPSV23), meningococcal conjugate (MenACWY), serogroup B meningococcal (MenB), Hib, annual influenza, hepatitis A and B, and HPV, provide essential protection against severe infections. Adherence to current immunization guidelines is paramount to safeguarding the health of individuals with SCA. While generally effective, the immune response to vaccines in SCA patients can vary, necessitating booster doses and additional vaccinations to ensure optimal protection. Overcoming challenges such as access to health care, vaccine hesitancy, and ensuring timely vaccinations are crucial to achieving high vaccination coverage in this population.

## Author contributions

**Methodology:** Emmanuel Ifeanyi Obeagu, Getrude Uzoma Obeagu.

**Supervision:** Emmanuel Ifeanyi Obeagu.

**Validation:** Emmanuel Ifeanyi Obeagu.

**Visualization:** Emmanuel Ifeanyi Obeagu.

**Writing—original draft:** Emmanuel Ifeanyi Obeagu, Getrude Uzoma Obeagu.

**Writing—review & editing:** Emmanuel Ifeanyi Obeagu, Getrude Uzoma Obeagu.
